# Genome-wide Analysis and Expression Divergence of the Trihelix family in *Brassica Rapa*: Insight into the Evolutionary Patterns in Plants

**DOI:** 10.1038/s41598-017-06935-0

**Published:** 2017-07-25

**Authors:** Wenli Wang, Peng Wu, TongKong Liu, Haibo Ren, Ying Li, Xilin Hou

**Affiliations:** State Key Laboratory of Crop Genetics and Germplasm Enhancement/Key Laboratory of Biology and Germplasm Enhancement of Horticultural Crops in East China, Ministry of Agriculture, Nanjing Agricultural University, Nanjing, 210095 China

## Abstract

Trihelix gene family is an important transcription factor (TF) family involved in plants’ growth and development. This extensive study of trihelix genes from *Arabidopsis thaliana* to *Brassica rapa* could shed light on the evolution in plants and support crop breeding. In this study, a total of 52 trihelix genes were identified in *B.rapa*. Whole-genome annotation, molecular-evolution and gene-expression analyses of all known trihelix genes were conducted. By statistics of the number of trihelix genes in each species, we found the expansion of trihelix gene family started with angiosperm evolution. And SIP1 was more preferentially retained than other subgroups (GT-1, GT-2, GT_γ_, SH4), consistent with the gene dosage hypothesis. Then we investigated the evolutionary patterns, footprints and conservation of trihelix genes in selected plants. The putative trihelix proteins were highly conserved, but their expression patterns varied. Half of these genes were highly expressed in all the selected organs but some showed tissue-specific expression patterns. Furthermore, among six abiotic stresses (Cold, Heat, PEG, NaCl, ABA and GA), most trihelix genes were activated by salt and ABA treatment. In summary, the phylogenetic, evolution and expression analyses of trihelix gene family in *B.rapa* establish a solid foundation for future comprehensive functional analysis of *BraTH*s.

## Introduction

The transcriptional regulation of genes plays important roles in both plant growth and in response to environmental stresses. Various classes of transcriptional factors (TFs) control the processes by interacting with cis-acting elements, or with other TFs involved in gene expression^1, 2^. Trihelix DNA-binding factors are a family of plant-specific transcription factor, which are classified as GT factors because they were discovered as proteins that bind specifically to GT elements^3–6^. The DNA-binding domain of GT factors features a typical trihelix (helix-loop-helix-loop-helix) structure. This is not a completely new domain as it has similarities to the individual repeats of the MYB family from which the trihelix may have been derived^5^. Taken together, with a degenerate core sequence of 5′-G-Pu-(T/A)-A-A-(T/A)-3′, the domain forms a specific binding site of GT elements^4, 6–8^.

Early studies suggested that trihelix factors are involved in regulating plant responses to light^4^. However, with more trihelix factors cloned and characterized in the past decade, this plant-specific transcription factor family has also been found to play important roles in a variety of developmental processes and stress responses, such as: morphogenesis control of manifold flower organs, seed scattering during crop domestication, responses to salt and drought stresses and the regulation of late embryogenesis^9–17^. *A. thaliana* was used to study most of the functions involved in plant development. The first discovered was the GT-1 factor of pea (*Pisum sativum*), which specifically binds to the light-induced gene *rbcS-3A* ^4^. Some other members of the GT-1 subfamily were later identified in rice, *Arabidopsis*, and tobacco^9, 18–20^. Recently, an important trihelix gene, *SHA1*, identified in rice, was found to be involved in regulating the seed scattering process^21^. Another two trihelix genes, *ASIL1* and *ASIL2*, have been reported to involve in chlorophyll accumulation in *A. thaliana* ^22^ (Table 1). Although most trihelix genes participate in plant developmental programs, two recent studies indicate that some are involved in plants stress-tolerance, especially salt tolerance^16, 17^ (Table 1). Loss-of-function mutations in *A. thaliana* GT-2 Like 1 (*AtGTL1*) gene, which negatively regulates water use efficiency by modulating stomatal density, led to increased plant tolerance to water deficit^23^. In addition, the involvement of two soybean trihelix factors [*GmGT-2A* (*Glyma04g39400*) and *GmGT-2B* (*Glyma10g30300*)] in abiotic stress tolerance has recently been proposed, following heterologous expression in *Arabidopsis* ^17^. Overexpression of these two genes could increase the tolerance to salt, drought, and cold. *OsGT*_*γ*_ *-1*, another gene found in γ clade, could regulate salt resistance with different expression level^16, 17^.

**Table 1:** The information of trihelix genes in *B. rapa* and known functions of *Arabidopsis* trihelix proteins (and those of related genes in other species).

	locus	Signature Domain		Pfam		Hit ID	Function	Refs
		start	end	Entry ID	E-value			
**GT-1**								
BraTH-28	Bra019721	81	162	PF13837	2.20E-21	AT1G13450.1		
BraTH-39	Bra026903	74	155	PF13837	7.10E-21	AT1G13450.1		
BraTH-47	Bra036354	75	154	PF13837	5.50E-19	AT3G25990.1		
BraTH-10	Bra005127	39	121	PF13837	2.70E-19	AT2G38250.1	Expression rapidly induced by salt, pathogen stress (Arabidopsis, soybean)	9–12
BraTH-01	Bra000046	44	126	PF13837	3.90E-20	AT2G38250.1		
BraTH-12	Bra005688	13	95	PF13837	3.60E-21	AT5G01380.1		
BraTH-42	Bra028899	2	62	PF13837	2.30E-14	AT5G01380.1		
BraTH-50	Bra038629	815	895	PF00753	0	AT5G63420.1	Lactamase/trihelix chimera, essential in early embryogenesis (Arabidopsis)	16, 17
**GT-2**								
BraTH-17	Bra008286	26	109	PF13837	4.60E-21	AT1G76890.2		
		358	443					
BraTH-25	Bra015715	43	127	PF13837	6.90E-21	AT1G76890.2		
		396	481					
BraTH-06	Bra003702	42	126	PF13837	6.50E-20	AT1G76890.2		
		340	425					
BraTH-26	Bra015716	58	142	PF13837	0	AT1G76880.1		
		366	451					
BraTH-07	Bra003703	53	137	PF13837	0	AT1G76880.1		
		408	493					
BraTH-16	Bra008285	54	138	PF13837	9.40E-18	AT1G76880.1		
		367	452					
BraTH-48	Bra036731	56	140	PF13837	9.60E-20	AT1G33240.1	Repression of endoreduplication in trichomes, repression of repressor of stomatal development, binds GGTAAA (Arabidopsis)	17, 22
		404	488					
BraTH-51	Bra040010	53	137	PF13837	2.30E-21	AT1G33240.1		
		388	472					
BraTH-20	Bra009994	101	182	PF13837	4.20E-11	AT5G28300.1	Tolerance to salt, freezing, drought stress (GmGT-2B,soybean)	17
		449	547					
BraTH-29	Bra020607	96	177	PF13837	1.60E-10	AT5G28300.1		
		451	547					
BraTH-37	Bra024925	257	323	PF13837	1.20E-06	AT5G47660.1		
BraTH-34	Bra022149	286	369	PF13837	1.10E-19	AT5G47660.1		
BraTH-19	Bra009518	114	199	PF13837	1.10E-20	AT5G03680.1	Regionalized growth suppression in developing perianth (Arabidopsis)	12
		404	490					
BraTH-41	Bra028824	123	208	PF13837	5.60E-21	AT5G03680.1		
		426	502					
BraTH-13	Bra005777	117	202	PF13837	1.20E-20	AT5G03680.1		
		407	492					
**GTγ**								
BraTH-38	Bra025881	89	183	PF13837	1.60E-24	AT1G21200.1		
BraTH-27	Bra016429	80	174	PF13837	2.30E-24	AT1G21200.1		
BraTH-08	Bra003704	66	150	PF13837	2.20E-18	AT1G76870.1	Tolerance to salt stress (rice)	16, 24
BraTH-44	Bra029813	110	214	PF13837	3.90E-20	AT3G10040.1		
BraTH-03	Bra001290	101	215	PF13837	2.80E-19	AT3G10040.1		
**SH4**								
BraTH-36	Bra023175	19	109	PF13837	3.90E-13	AT1G31310.1		
BraTH-23	Bra014906	19	108	PF13837	8.30E-14	AT1G31310.1		
BraTH-33	Bra021860	48	141	PF13837	0	AT2G33550.1		
BraTH-11	Bra005486	49	142	PF13837	0	AT2G33550.1		
BraTH-21	Bra010246	20	98	PF13837	4.80E-08	AT4G31270.1		
**SIP1**								
BraTH-18	Bra009119	32	113	PF13837	0	AT5G05550.1		
BraTH-46	Bra034866	25	107	PF13837	1.30E-25	AT3G11100.1		
BraTH-45	Bra034165	24	106	PF13837	1.20E-24	AT3G11100.1	A. tumefaciens 6b-interacting protein (tobacco)	19
BraTH-05	Bra003346	27	122	PF13837	2.10E-23	AT3G58630.1		
BraTH-15	Bra007407	26	127	PF13837	1.10E-23	AT3G58630.1		
BraTH-32	Bra021536	65	150	PF13837	2.70E-22	AT3G14180.1	Repression of late embryogenesis genes (Arabidopsis)	15, 22
BraTH-31	Bra021534	63	147	PF13837	3.10E-22	AT3G14180.1		
BraTH-49	Bra037960	73	155	PF13837	8.20E-23	AT1G54060.1	Repression of late embryogenesis genes, binds GTGATT (Arabidopsis)	
BraTH-22	Bra014370	75	161	PF13837	1.30E-22	AT1G54060.1		
BraTH-04	Bra001951	93	174	PF13837	2.30E-16	AT3G24490.1		
BraTH-43	Bra029812	162	248	PF13837	4.80E-21	AT3G10030.1	Trihelix/aa-kinase chimera, vegetative development (Arabidopsis)	9
BraTH-30	Bra021072	49	101	PF13837	5.40E-11	AT2G44730.1		
BraTH-14	Bra007069	40	123	PF13837	7.20E-20	AT3G54390.1		
BraTH-52	Bra040110	33	122	PF13837	7.10E-21	AT2G44730.1		
BraTH-09	Bra004859	43	134	PF13837	4.7E-20	AT2G44730.1		
BraTH-02	Bra000360	52	143	PF13837	2.70E-21	AT2G44730.1		
BraTH-24	Bra015090	62	142	PF13837	3.10E-21	AT3G24860.1		
BraTH-40	Bra028276	179	232	PF13837	1.80E-06	AT2G33550.1		
BraTH-35	Bra022563	147	229	PF13837	3.70E-08	AT2G33550.1		

The trihelix family had previously been classified into three distinctive subfamilies (GT_α_, GT_β_, and GT_γ_), using *Arabidopsis* and rice genes^24^. Then, Kaplan-levy *et al*. classified trihelix genes from rice and *Arabidopsis* into five clades, named GT-2, GT-1, SH4, SIP1, and GTγ, with the name of each clade based on the first member identified^9^.

The trihelix DNA-binding proteins are unique to plants, suggesting that they would be implicated in plant-specific gene regulation, as suggested for other plant lineage-specific factors^8^. There are 30 trihelix genes in *Arabidopsis* and 31 in rice. Compared with some of the big plant transcription factor families, such as the MYB, AP2/EREBP, NAC, and bHLH, all with more than 100 members in *Arabidopsis*, the number of trihelix genes is relatively modest^2^. Although trihelix genes have been identified in *Arabidopsis* and rice, the evolutionary and functional information of this family in Chinese cabbage are still unknown. Thus a more thorough systematic analysis is needed to uncover these mysteries.

The Chinese cabbage genome (Chiifu-401–42) has recently been sequenced and assembled^25^. Data suggested *B. rapa* was closely related to *A. thaliana*, and has experienced a whole genome triplication since its divergence from *A. thaliana* ^26, 27^. In this work, for distinguishing trihelix genes from different clades, they were abbreviated as TH. We systematically and comprehensively describe the TH transcription factors in *B. rapa* through a comparative genome analysis. The main objectives of our study were as follows: (i) identify and characterize the TH transcription factors in the *B. rapa* genome; (ii) analyze the copy number variation of trihelix genes and expansion following WGT in *B. rapa*; (iii) investigate the evolution of the trihelix gene family in the plant kingdom and construct its evolution model; (iv) construct TH transcription factor interaction networks, and analyze TH transcription factor expression patterns through comparative genomics.

## Results

### Identification of Trihelix proteins in plants and comparative analyses

**Figure 1:**
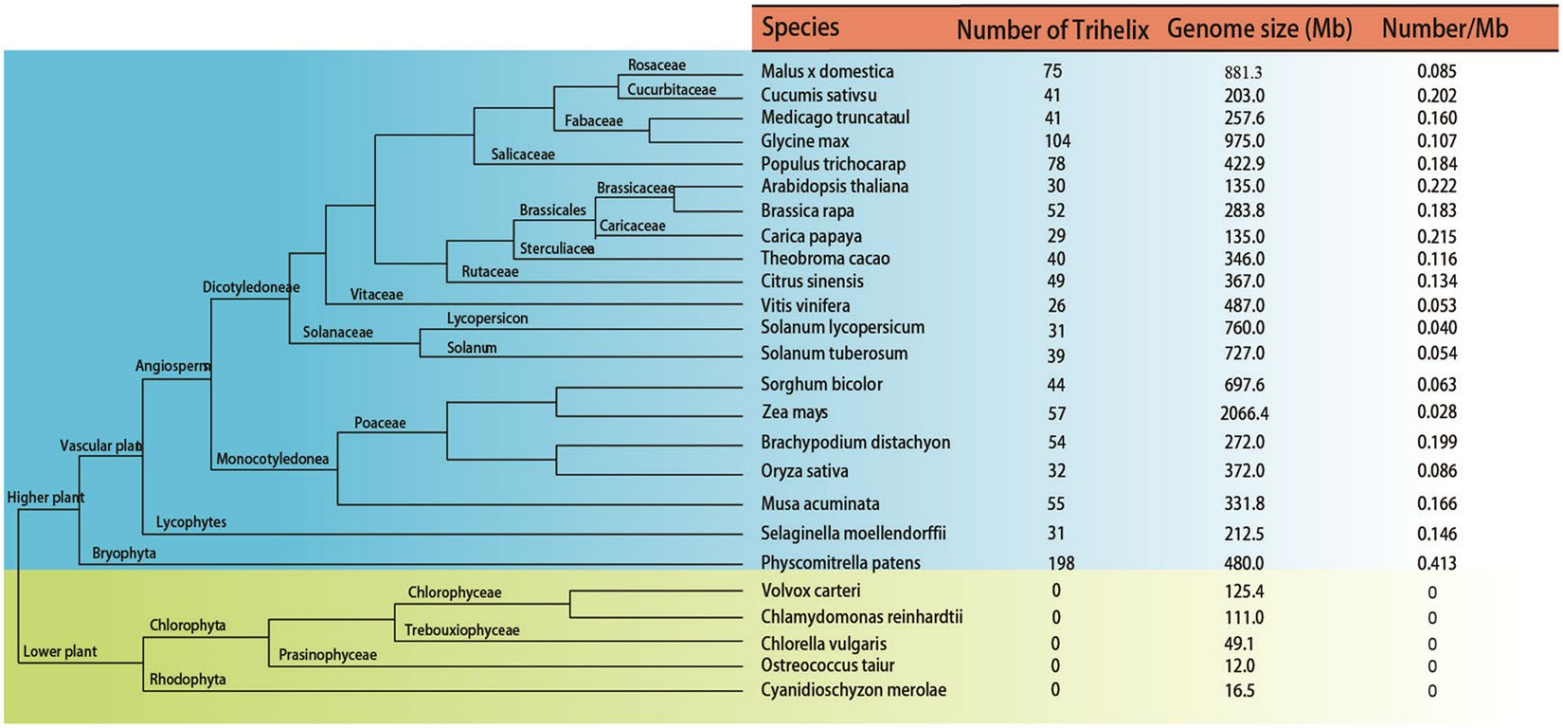
The relationships of the species and the number detail of the trihelix family of each species. The *left* of this figure shows the categories of the species; the *right* of this figure shows the number detail of the trihelix family of each species

We identified all the putative trihelix genes in *B.rapa* through HMM search. This search resulted in the identification of 52 trihelix proteins. Subsequently, all these protein sequences were subjected to Pfam and SMART analyses, and named *BraTH01* to *BraTH52* (Supplementary Table 2). For comparative genomic analyses, we searched for trihelix protein coding sequences in the representative genomes of 25 plants (Fig. 1) and identified a total of 1106 trihelix proteins (Supplementary Table 5). These proteins represent the major evolutionary lineages of the species for the analysis of the trihelix transcription factors. Interestingly, all of those transcription factors were only found in higher plants, none of them were found in lower plants. This phenomenon shows that the trihelix proteins may have expanded after the divergence of the higher plant from the lower plant species, and strongly suggests that this family is land plant-specific, consistent with previous studies.

Cumulatively, the number of trihelix genes in *B.rapa* (52) exceeded that in most other plants in our analyses. In terms of the density of trihelix proteins in the whole *B.rapa* genome (0.183), we found that it was more than that in most species used in our analyses. Although *Glycine max* contained 104 trihelix proteins, its trihelix protein density (*G. max*, 0.055) was lower than that in *B.rapa* due to its large genome size. This suggested that the trihelix proteins might play a very important role in plant evolution. Since several whole-genome duplication (WGD) events happened during angiosperm evolution, it is likely that this higher number is caused by an elevated duplication frequency, in combination with an increased retention of trihelix genes. Thus, the number and density of trihelix proteins increased as plants evolved, possibly because of genome duplication.

### Copy number variation and collinearity analysis of Trihelix genes

**Figure 2:**
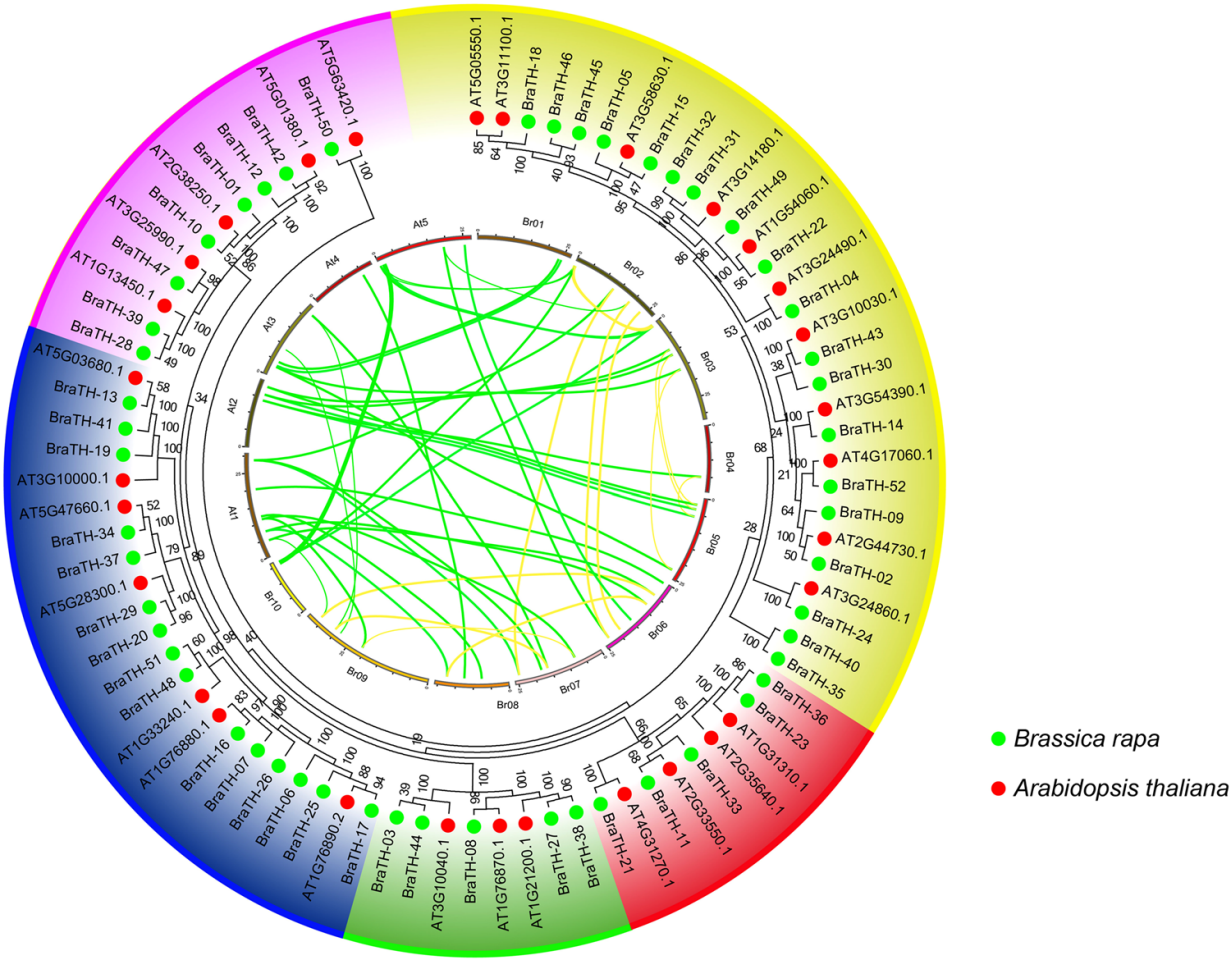
The syntenic trihelix genes between *Brassica rapa* and *Arabidopsis thaliana*. The ten Chinese cabbage chromosomes (Br01-Br10) and the five *A. Thaliana* chromosomes (At1-At5) are shown in different random colors. The green lines represent the syntenic genes pairs between Chinese cabbage and *Arabidopsis*, the yellow lines represent the syntenic genes in Chinese cabbage.

We then investigated the copy number variation of trihelix genes in *A. thaliana* and *B. rapa* during the Brassica-specific WGT event. There are 30 trihelix genes identified in *A. thaliana* and 52 in *B. rapa* (*BraTH01* to *BraTH52*) (Table 1 and Supplementary Table 2). The collinear relationships of the gene pairs in the Trihelix family in *B.rapa* are shown in Fig. 2. We totally identified 15 pairs (pairs and groups of three) of highly similar orthologous that shared a high degree of identity through the BRAD database. The *B.rapa* genome was divided into three sub-genomes according to their fractionation degree, namely the least fractionated (LF), medium fractionated (MF1), and most fractionated (MF2). In this study, 45 (87%) trihelix genes were identified in the three *B. rapa* sub-genomes and located in the syntenic regions (Fig. 2, Supplementary Fig. 1a and Supplementary Table 3). Then, we specifically compared the retention of trihelix genes by counting the number of gene copies and the different distributions of the three sub-genomes. It was found that the majority of SIP1 (22%) genes were retained in two or three copies, which is higher than the retention of other subfamily trihelix genes (Supplementary Fig. 1b).

### Expansion and Structural Characteristics of Trihelix Genes in Brassica rapa

**Figure 3:**
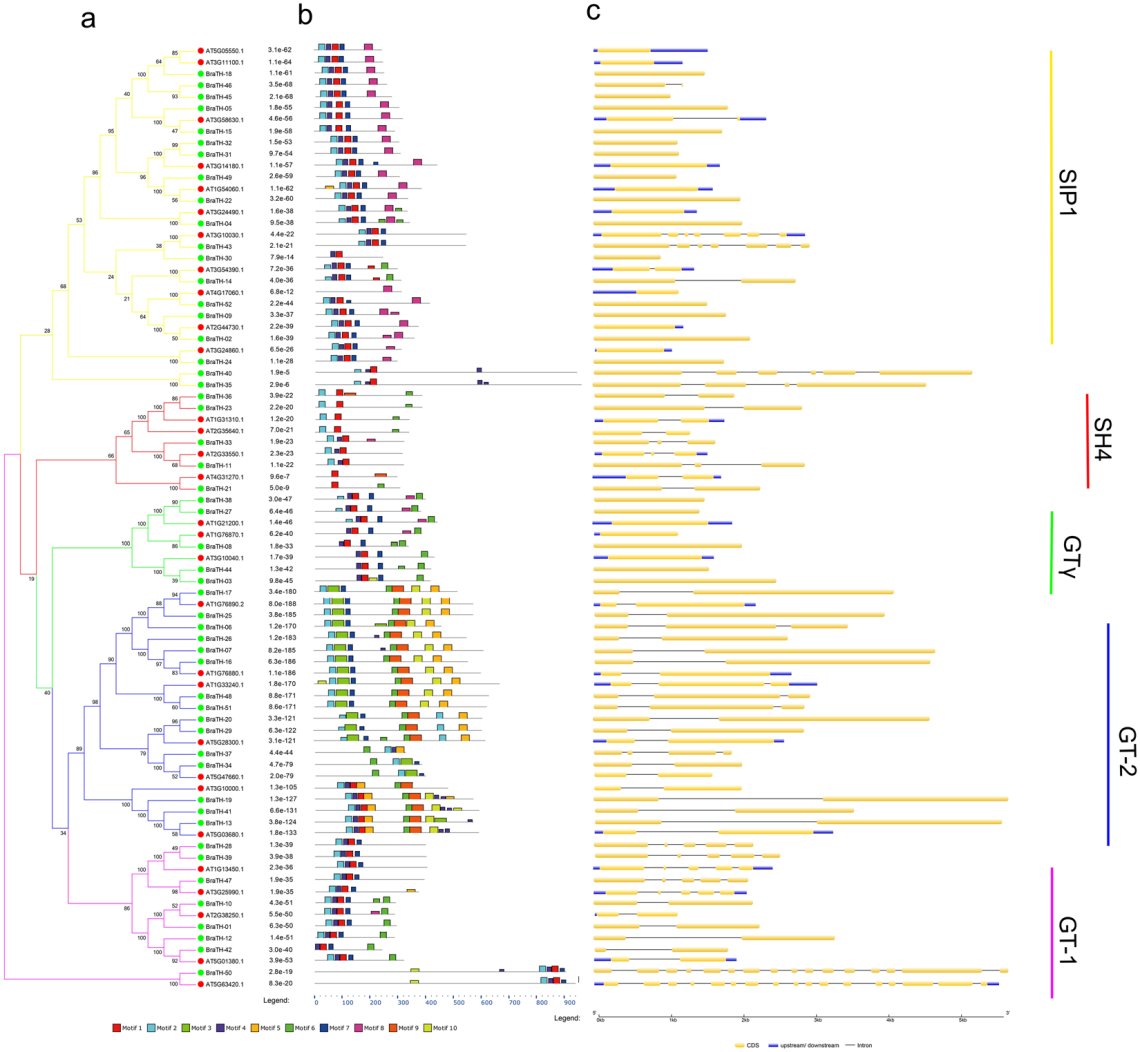
Schematic diagram of amino acid motifs and gene structures of trihelix genes in *Arabidopsis* and *B. rapa*.

To verify the extent of the lineage-specific expansion of the trihelix genes in *B. rapa* and *A. thaliana*, we performed a joint phylogenetic analysis of all the trihelix genes, and the homologous genes were marked on the tree (Fig. 3). All the trihelix genes were divided into five groups (SIP1, SH4, GTγ, GT-1 and GT-2), consistent with the previous reports in *A. thaliana* ^9^. Overall, almost no *GT-2s* was lost. After the split, *B. rapa* gained 9 and 1 genes and lost 8 and 4 genes in classes SIP1 and GTγ, respectively, resulting in the different expansion of these trihelix genes. Because of the Brassica-specific WGT event, the gene number of these two classes in *B. rapa* was greater than that in *A. thaliana*. (Fig. 3 and Supplementary Table 3).

**Figure 4:**
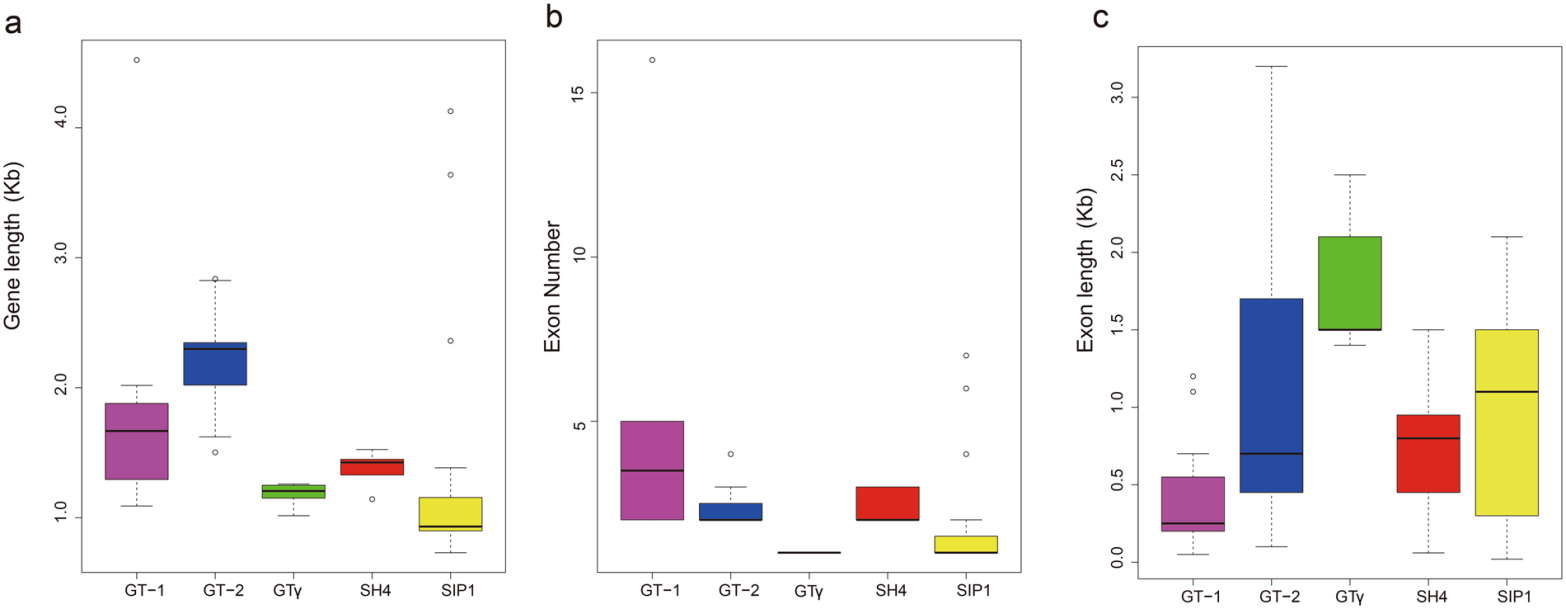
Boxplot of the gene length (**a**) exon numbers (**b**) and exon length (**c**) of the trihelix genes in *Arabidopsis* and *B. rapa*.

Furthermore, the sequence features of *B. rapa* trihelix proteins were also analyzed through MEME program, which can predict the conserved motifs among the *B.rapa* and *Arabidopsis* trihelix proteins. We identified 10 motifs in each comparison and named motif 1 to motif 10 (Fig. 3). Besides, the LOGO of these protein motifs was also obtained by MEME (Supplementary Fig. 2). Trihelix proteins often have similar motifs and intron/exon structure if they belong to the same group. All of the *BraTHs* contain motif 1, 2, 7, indicating that they all have a highly conserved domain. Additionally, besides the common motifs, 22 GT-2 clade trihelix members contain several specific motifs, such as motif 6, 10 that were shared in this subgroup. Interestingly, by comparing the genomic and cDNA sequences, we found that all the GTγ genes just have one exon and do not have intron, which is different from other clades. The average exon length of GTγ is greater than that of the SIP1, SH4, GT-1 and GT-2 (Fig. 4c). Furthermore, the number of exons in GTγ is the fewest, and in GT-1 is the most (Fig. 4b). Notably, the gene length of SIP1 was shorter but the exon length was longer than other subgroups (Fig. 4b,c).

### Chromosome distribution, Ks and duplication Analysis of the Trihelix Genes in B.rapa

All *BraTH* genes were positioned on the ten *B.rapa* chromosomes with a non-random distribution (Supplementary Fig. 3). Chromosome 07 and Chromosome 02 each contains the most *BraTH* genes (22%), whereas chromosomes 01, 04 and 10 each contains the fewest (4%) (Supplementary Fig. 3b). Additionally, according to the previous reports, we reconstructed the 24 conserved chromosomal blocks (labeled A–X) in *B. rapa* genome and the color coding of these blocks depended on their positions in a proposed ancestral karyotype (AK1–8)^25, 28^. AK1 and AK3 each contains most of the *BraTH* genes (21%), followed by AK6 (13%), while AK7 contains only 4% of *BraTH* genes (Supplementary Fig. 3c). Specifically, we also observed that some *BraTH* genes clustered together in a region of the chromosome. For example, 4 genes clustered in the end of chromosome 9, and two of them belonged to SIP1clade (*BraTH14* and *BraTH15*).

Furthermore, the duplication types were identified by the MCScanX program and the divergence time of the duplicated genes were estimated by calculating the number of synonymous substitutions (*Ks*) and *Ka* (nonsynonymous substitution rates). A total of 22 trihelix duplicated gene pairs were analyzed (Supplementary Table 4). SIP1, SH4, GTγ, GT-1 and GT-2 duplicated gene pairs belonged to segmental duplication, and all the duplicated *BraTH* gene pairs had a *Ka/Ks* ratio less than 1, indicating the purifying selection of these genes. Ranging from 0.3 to 0.5 and focusing on approximately 0.34 (~11 Myr), the *Ks* values of the *BraTH* genes were used to estimate the divergence time (Supplementary Fig. 4).The divergence time of *BraTH* duplicated gene pairs was 8 MYA, which indicates that their divergence occurred during the Brassica triplication events (5~9 MYA).

### Evolution footprint of Trihelix genes in plants

**Figure 5:**
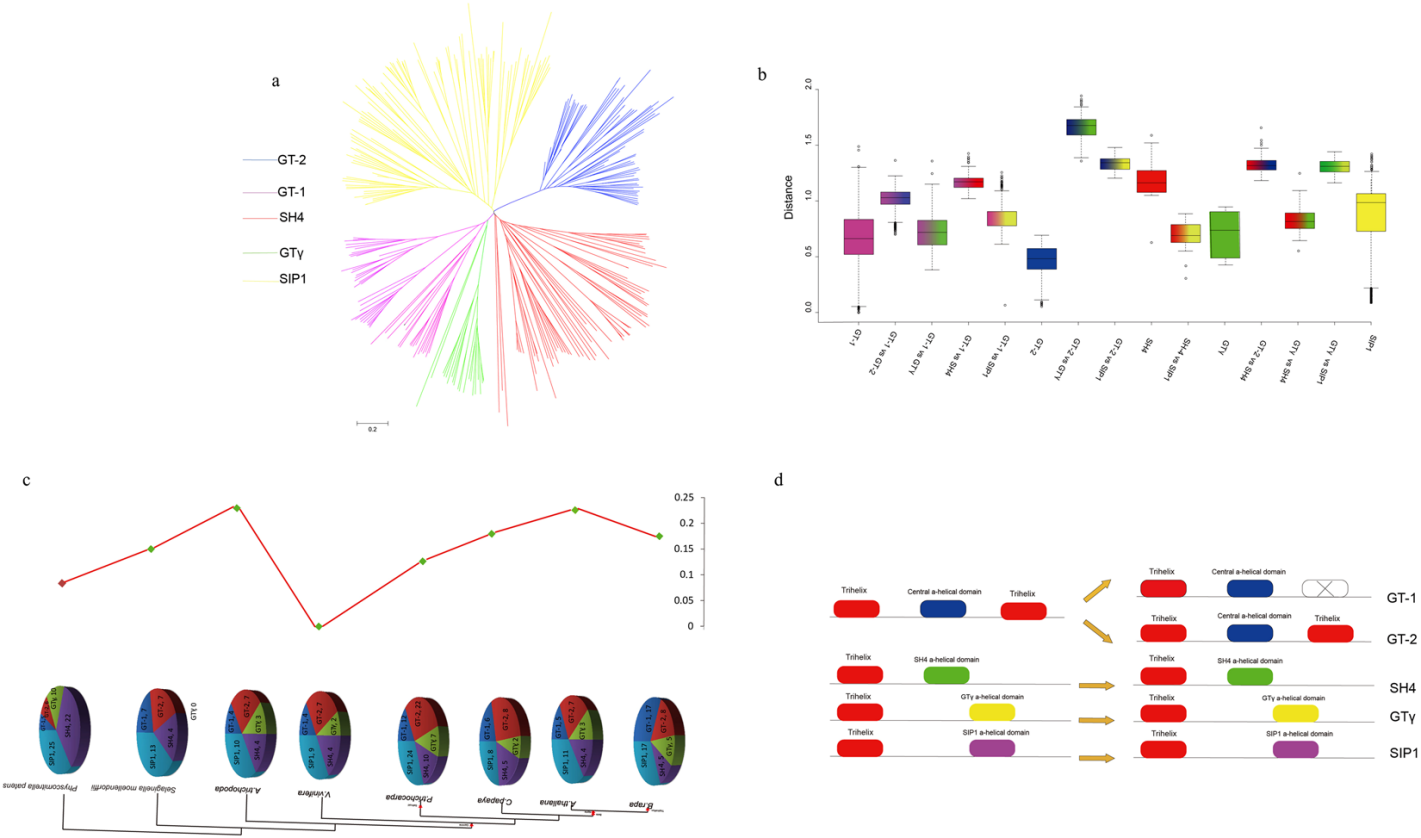
The analysis of trihelix genes evolution (**a**)Phylogenetic relationships among trihelix genes; (**b**) genetic distance among the different groups of trihelix genes; (**c**) comparison of the percentage of trihelix genes and copy numbers of trihelix genes, trihelix genes in representative species. (**d**) The rounded red box represents the trihelix domain, the rounded bule box represents the Central a-helica domain, the rounded green box represents the SH4 a-helica domain, the rounded yellow box represents the GTγ a-helica domain, the rounded purple box represents the SIP1 a-helica domain and the noncolored rounded box represents the lost domain.

To investigate the evolution of the trihelix gene family in the plant kingdom, we selected 8 representative plant species (*Brassica rapa*, *Arabidopsis thaliana*, *Carica papaya*, *Populus trichocarpa*, *Vitis vinifera*, *Amborella trichopoda, Phscomitrella. patens* and *Selaginella. moellendorffii*) for comparative analysis (Fig. 5). The reason is that *V. vinifera, P. trichocarpa*, and *C. papaya* did not undergo α and β duplications and *A. trichopoda*, a basal angiosperm, did not undergo the γ duplication event^29–33^. We constructed 8 phylogenetic trees of the trihelix genes to analyze the evolutionary relationships of these species (Supplementary Fig. 5). The phylogenetic trees showed that the trihelix gene family formed five distinct clades (SIP1, SH4, GTγ, GT-1 and GT-2), which is consistent with the result for *B. rapa* and *A. thaliana*. Trihelix genes were found exist in *A. trichopoda*, which indicates that these five groups originated from duplication events prior to the γ event. Meanwhile, no GTγ were detected in *S. moellendorffii*. Furthermore, we found that there were more trihelix genes existing in *P. trichocarpa* and *B. rapa* than in other species.

To further determine the relationship among the five subgroups, the analysis of genetic distance was performed with the box plot (Fig. 5b). It was shown that the genetic distance between GT-1 and GTγ was shorter than GT-1 with other groups (Fig. 5b). Notably, the genetic distance between the SIP1 and SH4 was shorter than that between the SH4 and GT-2, SH4 and GTγ, SH4 and GT-1. These results indicated that SH4 has a closer relationship with SIP1, which means SIP1 and SH4 may share a common evolutionary origin. Subsequently, the family size and the percentage of trihelix genes in eight plant species suggested that trihelix genes expanded rapidly during evolution and further expanded in the *Brassicaceae* (Fig. 5c). WGD is known to have significant impact on the expansion and evolution of gene families in plant genomes. However, along with the gradual increase in the trihelix percentage, the genes of GTγ were completely lost in *S. moellendorffii* (Supplementary Fig. 5). During the course of evolution, the expansion of SIP1 was relatively more stable when compared with other subgroups, and it appeared most recently and expanded most rapidly. Here, we proposed a possible evolutionary footprint or model of the trihelix gene family in plants (Fig. 5d). GT-2 contains two trihelix domains and one central a-helical domain. GT-1 is related to GT-2 but possess only one trihelix domain and one entral a-helical domain, possibly originated from GT-2 by losting one trihelix domain during the evolution; alternatively, it might be that GT-2 originated from GT-1 by gaining one trihelix domain.

### Tissue-specific expression Trihelix genes in Brassica rapa and *Arabidopsis Thaliana*

**Figure 6:**
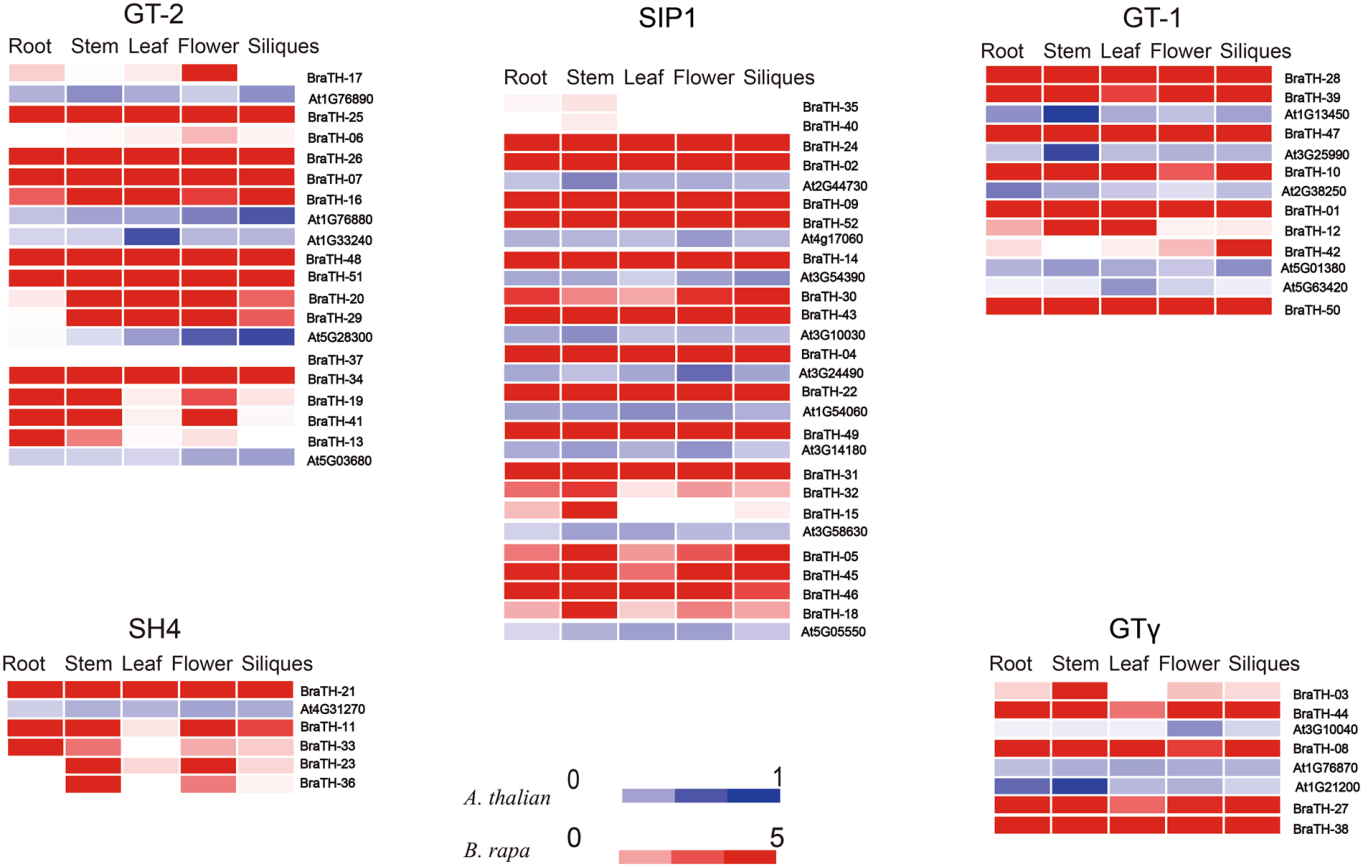
Analysis of the trihelix Genes in Different Tissues of *Brassica rapa* and *Arabidopsis* Heat map representation and hierarchical clustering of SIP1, SH4, GTγ, GT-1 and GT-2 genes in root, stem, leaf, flower and silique.

Since no trihelix factors in *B.rapa* has been previously documented, and to investigate the divergence of homologs and putative functions of trihelix genes in *B.rapa* growth and development, we analyzed the expression patterns of trihelix genes in five tissues (roots, stems, leaves, flowers, and siliques) of *A. thaliana* and *B. rapa* (Fig. 6 Supplementary Tables 6, 7). The results showed high alterations in expression levels among different TH group genes in *B.rapa*. Among 75 trihelix genes (including 23 *AtTH*s and 52 *BraTH*s), 1 (*BraTH37*) has no expression and 2 (*BraTH35* and *BraTH40*) have slight expression in any tissues. The rest of *AtTH*s and *BraTH*s were expressed in at least one tissue. Many proteins did not show striking differences in their expression levels among different organs or tissues. Half of (26) *BraTH* genes were highly expressed in all the five tissues and most of them belong to SIP1 subfamily. However, a small number of genes were detected selectively expressed highly in a specific tissue. Among them, 4 genes (*BraTH03, 15, 18, 36*) showed preferential expression patterns in the stem. Similarly, *BraTH17* predominantly expressed in the flower, whereas *BraTH42* has a relatively high expression level in the siliques (Fig. 6). Therefore, these genes may mainly function in organ- or tissue-specific development in *B.rapa*. Interestingly, several homologs showed highly similar expression patterns in five tissues. Meanwhile, most *BraTH* genes presented quite different expression profiles to their homologs in *Arabidopsis*. For instance, *At1G13450* and *At3G25990* had higher expression in stem than that of other organs (Fig. 6), whereas *BraTH28, 39, 47* were constitutively expressed in nearly all the organs with high abundance. The divergences in expression profiles between homologs revealed that some of them may acquire new functions after duplication in the evolutionary process.

We next investigated the expression trends among 13 duplicated gene groups (Supplementary Fig. 6). These duplicated genes showed different expression pattern types in five tissues. Seven pairs of duplicated *BraTH* genes were expressed in the same trend, suggesting that duplicated genes might have similar functions. Among them, *BraTH45/46*, *BraTH23/36*, *BraTH35/40* and *BraTH05/15* had an expression peak in steam, whereas *BraTH11/33* and *BraTH13/19/41* had the highest expression in root, besides, *BraTH28/39* was high expressed in flower (Supplementary Fig. 6c,g,h,m,d,j,f). Although they had similar expression trend, the expression abundance was differentiated. The duplicated gene groups are highly similar in their amino acid and nucleotide sequences, but that does not mean they all have the same expression trend and they may not be involved in the same pathway or do not have similar functions. For example, *BraTH12/42* showed a totally contrary expression trend and *BraTH01/10* exhibited relatively high transcript abundance in the stem and flower, respectively. Notably, *BraTH17/26/07/16/06/25* exhibited different transcript abundance in all the five tissues (Supplementary Fig. 6a). These two types of expression patterns suggested that the functions of these duplicated genes might have diverged in the course of evolution.

### Expression profiling and Coregulatory Networks of Trihelix genes in response to abiotic stresses and hormone

**Figure 7:**
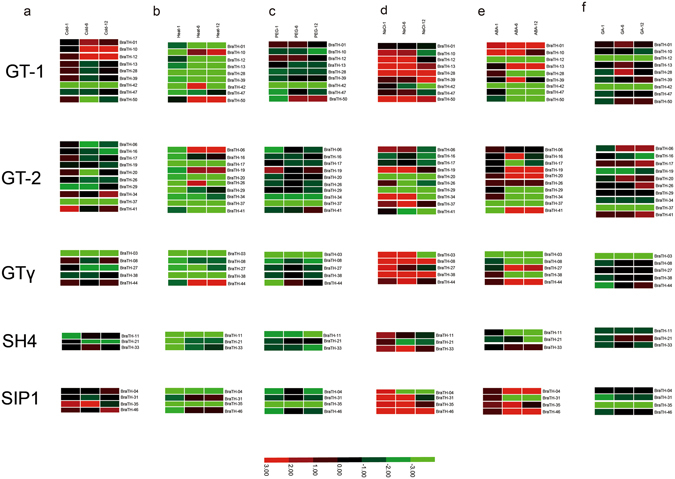
Expression analysis of *BraTH* Genes under six abiotic stress treatments. (**a,b,c,d,e,f**) Heat map representation the trihelix genes under six stress treatments, that is, Cold, Heat, PEG, NaCl, ABA and GA.

The examination of trihelix genes in function is now at an accelerating pace but the full functions of this family may not yet have been uncovered. Table 1 showed scattered examples of trihelix genes in responding to environmental stimuli. In recent two studies, some trihelix factors are reported to involve in the basic resistance to abiotic stresses, especially salt-resistence^16, 17^. To understand the expression profiles of trihelix genes under different environmental conditions, the expression patterns of 31 selected trihelix genes were studied in response to various abiotic stresses and hormone treatments using qRT-PCR experiment (Supplementary Table 8). Heat map representation for transcript expression fold change in response to abiotic stresses and hormone treatments was shown in (Fig. 7, Supplementary Fig. 8).

Under four abiotic stresses (cold, heat, PEG, NaCl), most of *BraTH* genes were upregulated by NaCl and downregulated by PEG treatment, just as previous reports in *Arabidopsis* (Table 1). Under the two hormone treatments (ABA and GA), more genes were induced by ABA treatment compared to the GA treatment. Meanwhile, the expression profiling of the five clades was also different from each other. It seems that the GT-1 and GT-2 clade genes are more sensitive to stresses. All the SH4 genes were downregulated after heat and PEG treatments and the majorities of GT-1(except *BraTH-10* and *BraTH12*) were downregulated after cold treatment. By contrast, the SIP1 clade was significantly induced in response to NaCl and ABA treatments and slightly induced by Cold treatment but showed repression after heat, PEG and GA treatments. In GT_γ_ clade, all the five *B.rapa* genes (except *BraTH03* and *BraTH27* at 12 h) (Fig. 7d), were significantly induced under NaCl treatment, as well as heat and ABA treatment. It is worth mentioning that some homologous genes among *B.rapa* and *Arabidopsis* showed quite different expression patterns under the same stress conditions. For example, it was reported that the *At5g28300* was induced by salinity, drought, cold and ABA in two-week-old seedlings (Table 1)^9^. However, we did not find its homologs (*BraTH20* and *BraTH29*) in *B.rapa* had the same expression pattern. There are three GT_γ_ group genes in *Arabidopsis* (Table 1)^24^, but their expression seems not show the similar trends^34^, and it will be of interest to further study the extent of stress-related functions. Regulatory subfunctionalization may have contributed to the transcriptional divergence among the genes in *B.rapa* and *Arabidopsis*, as it is not very likely these genes evolved all of the functions independently in chilling, hormone, and salt tolerance.

**Figure 8:**
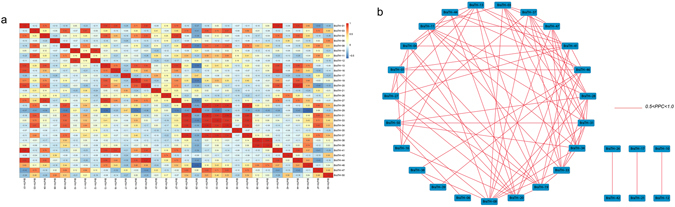
Correlations and co-regulatory networks of 31 trihelix genes under stress treatments. (**a**) Correlation analysis by using the R package program. Each correlation is shown by the shades of blue and orange. Blue and orange indicate a positive correlation and negative correlation, respectively. (**b**) Co-regulatory networks. The co-regulatory networks of 31 trihelix genes under stress treatments were established based on the Pearson correlation coefficients (PCC) of these gene pairs using transformed qPCR data The PCC of co-regulatory gene pairs was considered significant at the 0.05 significance level (p-value), and different colour line styles indicate the different significance levels of the co-regulated gene pairs.

To further understand the connection between these trihelix genes, we established the correlation and co-regulatory networks based on the PCCs of the relative expression of genes (Fig. 8, Supplementary Table 9). Some genes showed close correlations, such as *BraTH28* and *BraTH31* and *BraTH38* and *BraTH39*. Additionally, a number of genes exhibited inverse correlations, such as *BraTH29* and almost all of other *BraTHs*, except *BraTH10, 17, 21* (Fig. 8a). *BraTH* gene pairs with PCC values that were significant at the 0.05 significance level and were greater than 0.5 were collected and visualized to construct hormones and abiotic stresses coregulatory networks (Fig. 8b). All the gene pairs with positive significant correlations were shown in the co-regulatory network, a total of 29 nodes. A close relationship was observed between GT-1 genes and other subfamilies. Meanwhile, most of the duplicated genes seem to have no correlation, except *BraTH11/33* and *BraTH13/19/41*. The divergence trend of the duplicated genes was reflected by the networks. The networks depicted the expansion of the gene family, which could help plants adapt to the diversified living environment by increasing cooperation or obtaining new functions.

Moreover, to research how *BraTH* genes interact with other genes, an interaction network associated with *BraTH* genes was built according to *Arabidopsis* orthologs (Supplementary Fig. 8). The green and yellow lines stand for positive correlation (Pearson correlation coefficient >0) with 98 pairs of interacting genes, negative correlation (Pearson correlation coefficient <0) with nine pairs of interacting genes. The interaction network of *BraTH* genes showed a very complicated correlation with other genes in Chinese cabbage, which may indicate *BraTH* genes involve in many fundamental mechanisms by regulating many downstream factors or being regulated by many upstream genes.

### BraTH28 targeted to the nucleus

**Figure 9:**
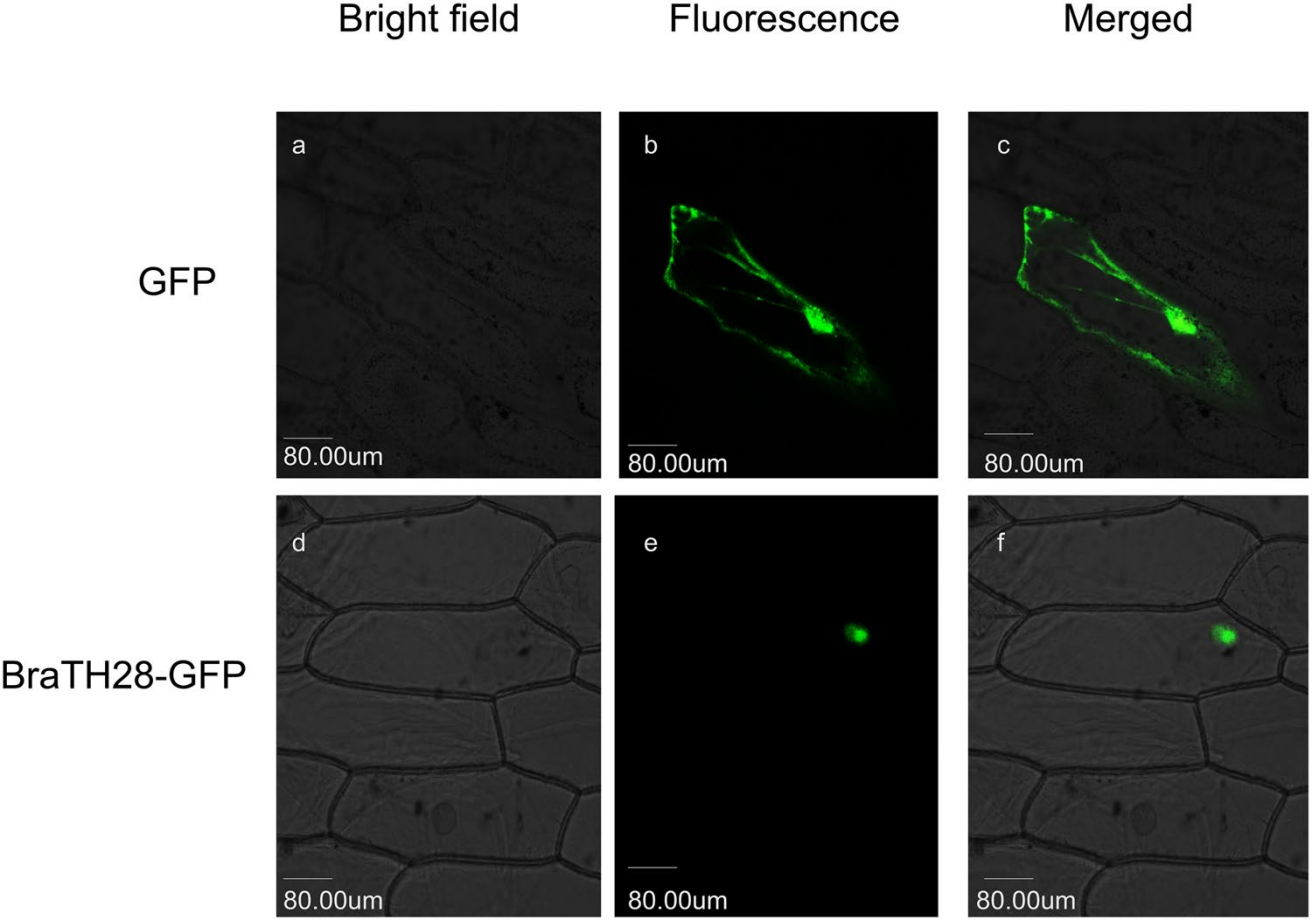
Nuclear localization of *BraTH28* in onion epidermal cells. Onion epidermal cells were transfected by 35S::GFP (**a–c**) or 35S::*BraTH28*-GFP (**d–f**) and photographed under a confocal microscope at 488 nm after 48 h (**a,d**). (**b** and **e**) are respectively the transmission image of (**a** and **d**). (**c** and **f**) are merged images of (**a** and **b** or **d** and **e**) respectively.

Sequence analysis showed the existence of a putative nuclear localization signal in *BraTH28*. To test whether *BraTH28* is targeted to the nucleus, a *BraTH28*-GFP fusion construct under the control of the CaMV 35S promoter was introduced into onion epidermal cells. The GFP alone is located throughout the cell, while *BraTH28* specifically targets the nucleus and possesses both transcriptional activation and DNA-binding abilities, implicating its function as a nuclear transcription factor (Fig. 9).

## Discussion

During the course of evolution, abundant genetic materials and bulk genetic variations have been provided by genome duplication, which supports plants to adapt better to diversified environments, such as drought, high salinity, and extreme temperatures. Transcriptional regulation of gene expression plays a major role in both plant development and in response to environmental stimuli. Trihelix transcriptional factors are involved, directly or indirectly, in diverse physiological processes associated with stresses, the development of perianth organs, trichomes, stomata and the seed abscission layer, and the regulation of late embryogenesis^11, 17–19^. In this study, 52 trihelix genes were identified in the *B. rapa* genome, and they contained a high number of gene copies. This finding suggests that these genes had a high degree of retention following WGD. Thus, the central issue in the evolution of duplicated genes is why *BraSIP1* were retained more than other subgroups. One possible explanation is that the functional requirement plays important roles in both plants’ developmental processes and defenses^35, 36^. This finding is consistent with the gene dosage hypothesis that genes encoding members of macromolecular complexes were preferentially retained following polyploidy and underrepresented in copy number variants, thus keeping the network stability^37, 38^. In addition, we found that *BraTHs* diverged 8 MYA during the Brassica-specific WGT event. We inferred that there may have been a stronger selective pressure on *BraTHs* that made them duplicate early to meet their survival needs, reflecting that the functions of *BraTHs* were more varied and complex.

In previous reports, the trihelix family is apparently limited to land plants^9^, although a report of their presence in humans and Drosophila^39^ needs to be studied further. They do not exist in the green algae (*Chlorophyta*)^40–42^, and have undergone large scale expansion in the lineage of the last common ancestor of land plants^42^. The presumed origin of the trihelix domain from a MYB-like gene carrying only one repeat^5^, and their relationship to other divergent MYB-like genes, needs to be examined in further detail. In this work, a phylogenetic tree of trihelix transcription factors from *B.rapa* and the dicotyledonous model plant *Arabidopsis* was constructed. The result was consistent with domain and Trihelix type classifications of *B.rapa* trihelix transcription factors. Basing on the current genomic data, we built a model diagram for the origin and evolution of trihelix family transcription factors. Among all motifs, motif 1, 2, and 4 contained a (F/Y)- (F/Y)-X-X-(L/I/M)-X-X-(L/I/M) sequence. Motif 9 and 10 were present in GT-2 members, while motif 1 and motif 4 was found in other subgroups. In addition, comparative structural analysis of *BraTHs* revealed that *BraTHs* in the same group shared similar exon–intron structures. The analysis on structures of *BraTH* genes may provide a way to find out which group of trihelix genes might be of a more ancient origin. The Ks values supported that trihelix genes did not have significant difference among the three subgenomes (LF, MF1, MF2). Actually, they may have similar gene structures, the similar intron and exon numbers of each subfamily also supported that. Taken all the results together, our study offers significant insights into the unique features and roles of this family in eukaryotic organisms. The fairly high conservation in gene structure observed here between genes identified by phylogenetic reconstruction was to be expected in genes of an ancient family which play a key metabolic role in virtually all living organisms.

**Figure 10:**
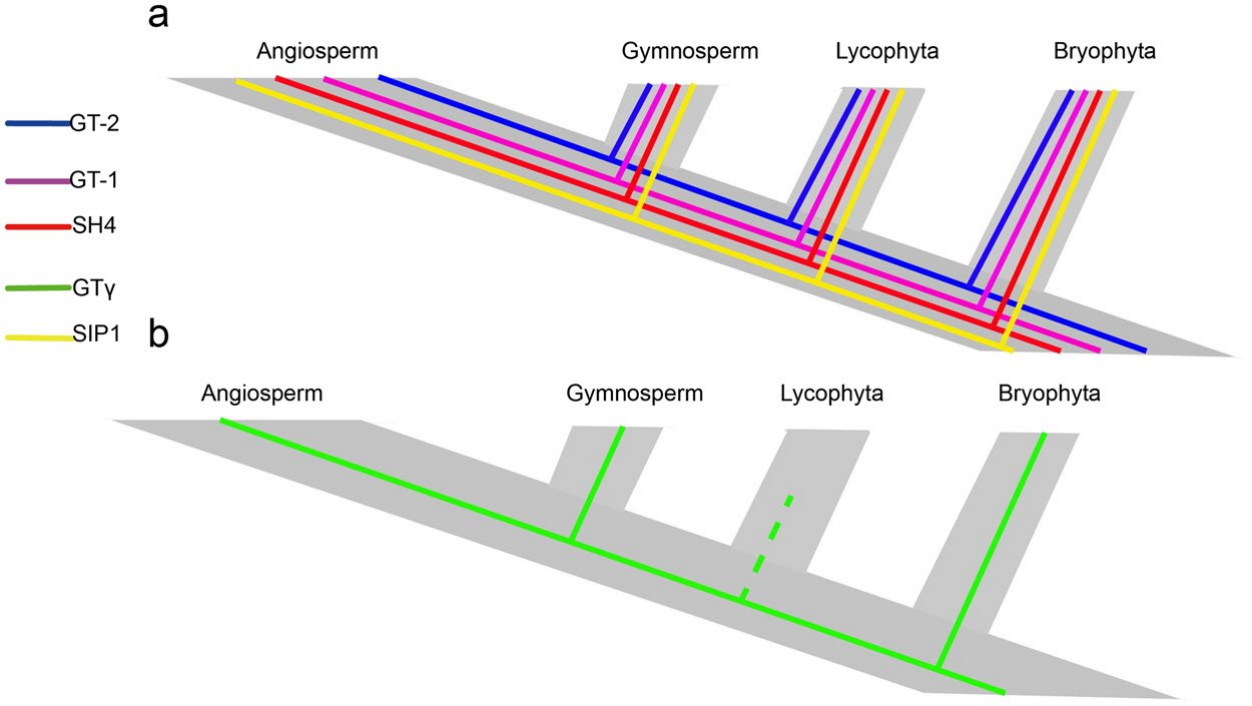
Evolutionary history of the trihelix family in plants. (**a**) GT-2, GT-1, SH4, SIP1 (indicated by different colors) exist in the course of evolution from Bryophyta to Angiosperm. (**b**) GTγ (indicated by green line) exists in the course of evolution from Bryophyta to Angiosperm except Lycophyta (dummy line indicates inexistence).

Most land plants have undergone polyploidization during their long evolutionary histories^43, 44^. Polyploidy not only led to WGD but also offered chances for duplicated genes to diverge subsequently in three broad evolutionary ways: subfunctionalization, neofunctionalization, and nonfunctionalization (pseudogenization or deletion)^44^. Some duplicated genes could also have completely redundant functions^45^. Through the analyses of (i) phylogenetic relationships, (ii) gene structures, (iii) synteny analysis and (iv) nucleotide distance, we found that SIP1 had a close relationship with SH4, and we constructed the evolutionary model of trihelix family (Fig. 5d). We estimated the cleavage trihelix domain mechanisms, of which the domain may be modified at the genome level. By comparative analysis in all selected plants, here, we inferred an evolutionary history of trihelix family in the plant kingdom: from Bryophyta to Angiosperm, all five clades exist from Bryophyta; then, GTγ was absent in Lycophyta while existed in angiosperm plants like other four groups (Fig. 10). All the data obtained are compatible with trihelix genes emerging very early in eukaryotic evolution and being transmitting both vertically and horizontally.

In addition to analyses of the evolutionary history of trihelix genes, based on the complete genome sequences and sequence similarities, we attempted to predict their functions in various species^46, 47^. Due to the similar intron and exon numbers, the homolog genes may have similar gene structures. Their highly conserved sequences were further proved by analyzing the proteins with MEME. In addition, both the duplicated genes in the neofunctionalization or subfunctionalization models and the expansions of the large gene family were associated with the processes of tissue-expression divergence^48–50^. In this study, the tissue-specific expression patterns of trihelix genes were also examined: most trihelix genes were highly expressed in all the five tissues or several at least. Meanwhile, a few of genes showed tissue-specific expression and some trihelix genes of different clades had similar expression patterns, indicating their common importance in plant development. The genes expressed in specific tissues might have acquired new functions related to plant development. The divergences in expression profiles between homologs revealed that some of them may acquire new functions after duplication in the evolutionary process.

In summary, it seems reasonable that repeated WGD events facilitated the increase in trihelix genes network complexity, such as in *A. thaliana* and *B. rapa* (Supplementary Fig. 8). By integrating phylogenetic, molecular evolution, gene structure and expression pattern analyses and conducting a comparative analysis with the currently available genome information in the selected plants (Figs 1 and 5), our study provides a deep understanding of the evolutionary history of trihelix gene family in plants. The evolution and origin of the trihelix genes in the plant kingdom were analyzed, and the evolutionary pattern of the trihelix genes was determined (Figs 5d and 10). Due to visible tissue-specific expression patterns, the expansion of trihelix genes seems to be correlated with the evolution of increasingly complex organs in plants. This finding will lead to novel insight into functional divergence and conservation in this gene family.

## Materials and Methods

### Identification of the Trihelix Genes in Multiple Species

All the *B. rapa* genome sequence data were downloaded from the Brassica database (BRAD; http://brassicadb.org/brad/)^27^. The protein sequences of *A. thaliana* trihelix were obtained from the *Arabidopsis* Information Resource database (http://www.arabidopsis.org/; Supplementary Table S1). The gene information of *Amborella. trichopoda* was obtained from the *Amborella* Genome Database (http://www.amborella.org/)^32^. The gene information of *Ca. papaya, V. vinifera, Po. trichocarpa, Ph.patens* and *S. moellendorffii* were downloaded from Phytozome v9.1 (http://www.phytozome.net/;)^51^. To identify putative trihelix family members, the Hidden Markov Model (HMM) profiles of trihelix (PF13837) were retained from the Pfam database (http://pfam.xfam.org/) and were used to identify the putative trihelix proteins with the best domain e-value cutoffs of <1 × 10^−4^. With a cutoff e-value of <10^−10^, the *Arabidopsis* trihelix sequences were used as the query to perform a BLASTP search. The SMART tool(http://smart.embl-heidelberg.de/) and the National Center for Biotechnology Information (NCBI) database (http://www.ncbi.nlm.nih.gov/) were used to analyze these potential sequences to validate the HMM and BLAST search^52^.

### Synteny Analysis

The Multiple Collinearity Scan toolkit (MCScanX) was used for the synteny analysis between the *A. thaliana* and *B. rapa* genomes according to previous reports. (http://chibba.pgml.uga.edu/mcscan2/; match_score: 50, match_size: 5, gap_score: −3, E_value: 1E–05)^53–55^. An all-against-all BLASTP comparison provided the pairwise gene information and P values for primary clustering. The whole-genome protein sequences from *B. rapa* and *A. thaliana*, were searched against themselves using BLASTP (E < 1e-10, identity >75%). Further, MCScanX was also used to identify WGD/segmental, tandem, proximal and dispersed duplication events in the *BraTH* family.

### Ks Analysis

The protein sequences of trihelix from *B. rapa* were aligned with their syntenic genes in *A. thaliana* using MUSCLE^56^. To estimate the divergence of the duplicated trihelix genes, the sequences of the duplicated pairs of trihelix genes were aligned using ClustalW2. We calculated the synonymous rate (Ks), non-synonymous rate (Ka), and evolutionary constraint (Ka/Ks).An in-house Perl script based on ParaAT54 was used to translate the protein alignments into coding sequence alignments and based on that we calculated the Ka (nonsynonymous substitution rate) and Ks (synonymous substitution rate) values using the method of Nei and Gojobori implementing in KaKs_calculator^57^. The Ks values were then used in the density and boxplot through the R3.3.0 program^58^. The formula T = Ks/2r was used to calculate the divergence time in which the r was taken to be 1.5 × 10^−8^ synonymous substitutions per site per year, representing the rate of divergence^59^.

### Evolution Analysis of trihelix Gene Family

The MUSCLE program was used to align the full-length trihelix proteins sequences with the default parameters^56^. The maximum-likelihood method was used to construct the phylogenetic relationship in each analysis. MEGA5.2 was used to calculate Bootstrap values with 1,000 replications^60^. To estimate the nucleotide divergence between sequences, all nucleotide sequences of the trihelix genes were also analyzed with MEGA 5.2 using the Jukes-Cantor model. Bootstrap (1,000 replicates) analyses were also performed for this estimation.

### Motif Identification and Exon–Intron Structural Analysis

To identify the conserved motifs of the trihelix genes of *B. rapa*, the online Multiple Expectation-maximization for Motif Elicitation program version 4.9.0^61^ was employed among the amino acid sequences with the default parameters, except for the following parameters: Maximum number of motifs, 10; optimum motif width 20 and 120. The position information of the trihelix genes, and trihelix domains was obtained from the Pfam database, and the information of gene structure was obtained from the General Feature Format files. We then draw the domain and exon–intron structures positions through the online program GSDS (http://gsds.cbi.pku.edu.cn/)^62^.

### Expression Pattern Analysis for Trihelix Genes in Five Tissues

For expression profiling of the trihelix genes in *B. rapa*, we analyzed five tissues of *B. rapa* accession Chiifu-401-42 (root, stem, leaf, flower, and silique). The Illumina RNA-seq data which were previously generated and analyzed by^25^ were utilized. Gene expression patterns of each tissue were analyzed and fragments per kilobase of exon model per million mapped (FPKM) values were log2 transformed. The gene expression patterns of each tissue were analyzed using Cluster 3.0, and the expression values were log2 transformed. Finally, heat maps of hierarchical clustering were visualized using Tree View (http://jtreeview.sourceforge.net/). The *A. thaliana* development expression profiling was analyzed using the AtGenExpress Visualization Tool (AVT; http://jsp.weigelworld.org/expviz/expviz.jsp) with mean-normalized values. Heat maps of the gene FPKM values in *B. rapa* and *A. thaliana* were visualized using Tree View (http://jtreeview. sourceforge.net/).

### Plant Materials

The Chinese cabbage cultivar Chiifu-401-42 was used for this experiment. This cultivar is a typical cultivar for Chinese cabbage research as its whole genome sequencing has been completed. Seeds were surface sterilized in 12% sodium hypochlorite before germinating on 0.5 Murashige and Skoog (MS) agar plates (0.7%) in a growth chamber at 22 °C in the dark for 2 days. The germinated seeds were grown in pots containing a soil: vermiculite mixture (3:1) in the greenhouse of Nanjing Agricultural University, and the controlled environment growth chamber was programed for 75% humidity, light 16 h/25 °C and dark 8 h/20 °C. One month later, the five-leaf stage seedlings were transferred to 4 or 38 °C growth chambers under the same light intensity and day length as the cold and heat treatments. Pots were irrigated with 250 mM NaCl and 15% (w/v) polyethylene glycol (PEG) for 30 min under normal growth conditions as salt and osmotic treatments, respectively. Simultaneously, some plants were grown in 1/2 Hoagland’s solution in plastic containers with the pH at 6.5 for acclimation. 5 days later, plants were grown in the following three treatments: (1) Control; (2) 100 μM GA; (3) 100 μM ABA; We sampled at 1, 6 and 12 h, the young leaf samples were frozen in liquid nitrogen and stored at −70 °C for further analysis.

### RNA Isolation and qRT-PCR analyses Analysis

The RNA was isolated from leaves using an RNA kit (RNAsimply total RNA Kit; Tiangen, Beijing, China) according to the manufacturer’s instructions. We used agarose gel electrophoresis to assess the quality and quantity of every RNA sample. The RNA was then reverse transcribed into cDNA using the Prime Script RT reagent Kit (TaKaRa). The Supplementary Table S1 listed the gene-specific primers used for real-time polymerase Chain Reaction (PCR). The reactions were performed using a Step one plus Real-Time PCR System (Applied Biosystems, Carlsbad, CA). The PCR parameters were as follows: 94  °C for 30 s, 40 cycles at 94 °C for 10 s, and 60 °C for 30 s, and then a melting curve (61 cycles at 65 °C for 10 s) was generated to check the specificity of the amplification. Relative fold expression changes were calculated using the comparative Ct value method^63^.

### Pearson Correlation Analyses

Based on log2-transformed quantitative Real-Time (qRT)-PCR data, we calculated Pearson correlation coefficients (PCCs) of transcript levels of trihelix gene pairs by R program. For a gene coregulatory network analysis, we collected the gene pairs whose PCC was more than 0.5 and significant at the 0.05 significance level (P -value). Based on the PCCs of these gene pairs, the co-expression networks were visualized through Cytoscape^64^. The interaction network associated with *Arabidopsis* orthologous of trihelix genes in Chinese cabbage was constructed using the *Arabidopsis* interaction viewer and cytoscape software^64^.

### Nuclear localization assays

The *BraTH28* coding region (without the stop codon) was amplified by PCR and inserted into the psmGFP vector^65^ to produce the *BraTH28*-GFP fusion construct. Onion epidermal cells were transfected by biolistic bombardment using the PDS-1000/He system (Bio-Rad) according to the manufacturer’s instructions and imaged with a confocal microscope (LSM 510, Zeiss).

## Electronic supplementary material

Supplementary Information

Dataset 1
