# Supplementary material for: Genome-wide Analysis and Expression Divergence of the Trihelix family in Brassica Rapa: Insight into the Evolutionary Patterns in Plants
Source: Sci Rep. 2017 Jul 25;7:6463. doi: 10.1038/s41598-017-06935-0 (PMC5526864; doi:10.1038/s41598-017-06935-0)
Supplement: Supplementary file 1 — Supplementary Information [file 41598_2017_6935_MOESM1_ESM.pdf]

Genome-wide Analysis and Expression Divergence of the Trihelix family in *Brassica Rapa*: Insight into the Evolutionary Patterns in Plants

Wenli Wang[1] , Peng Wu[1] , TongKong Liu[1], Haibo Ren[1] , Ying Li[1] , Xilin Hou[1*]

[1]State Key Laboratory of Crop Genetics and Germplasm Enhancement/Key Laboratory of Biology and Germplasm Enhancement of Horticultural Crops in East China, Ministry of Agriculture, Nanjing Agricultural University, Nanjing 210095, China

*To whom correspondence should be addressed: hxl@njau.edu.cn

Tel.: +86 025 8439 5917

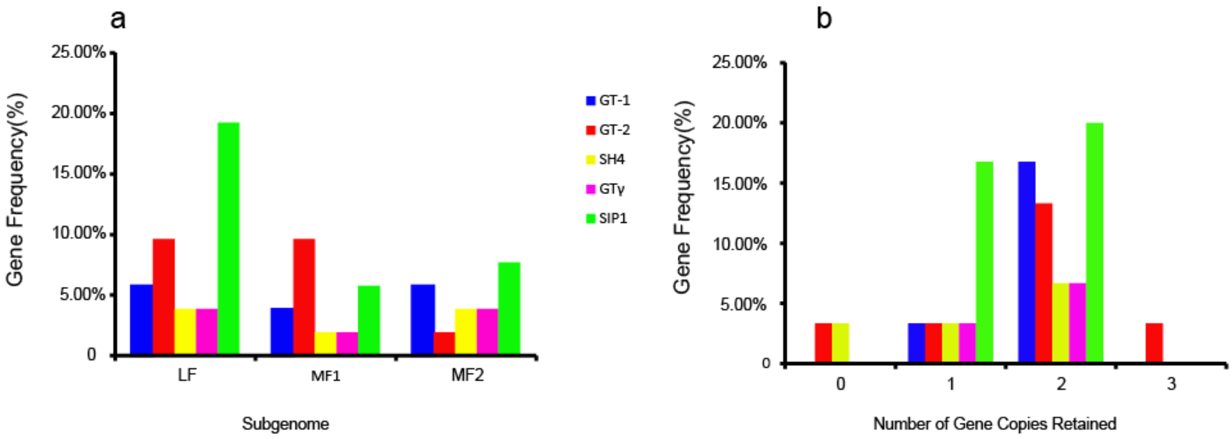

Supplementary Fig. 1 Copy number variation and differential retention of trihelix genes in *B.rapa*. **a** Retention of trihelix genes in the three subgenomes (LF, MF1, and MF2) in *B. rapa*. Least fractionated (LF), medium fractionated (MF1) and most fractionated (MF2) subgenomes. **b** Copy numbers of trihelix genes after genome triplication and fractionation in *B. rapa*.

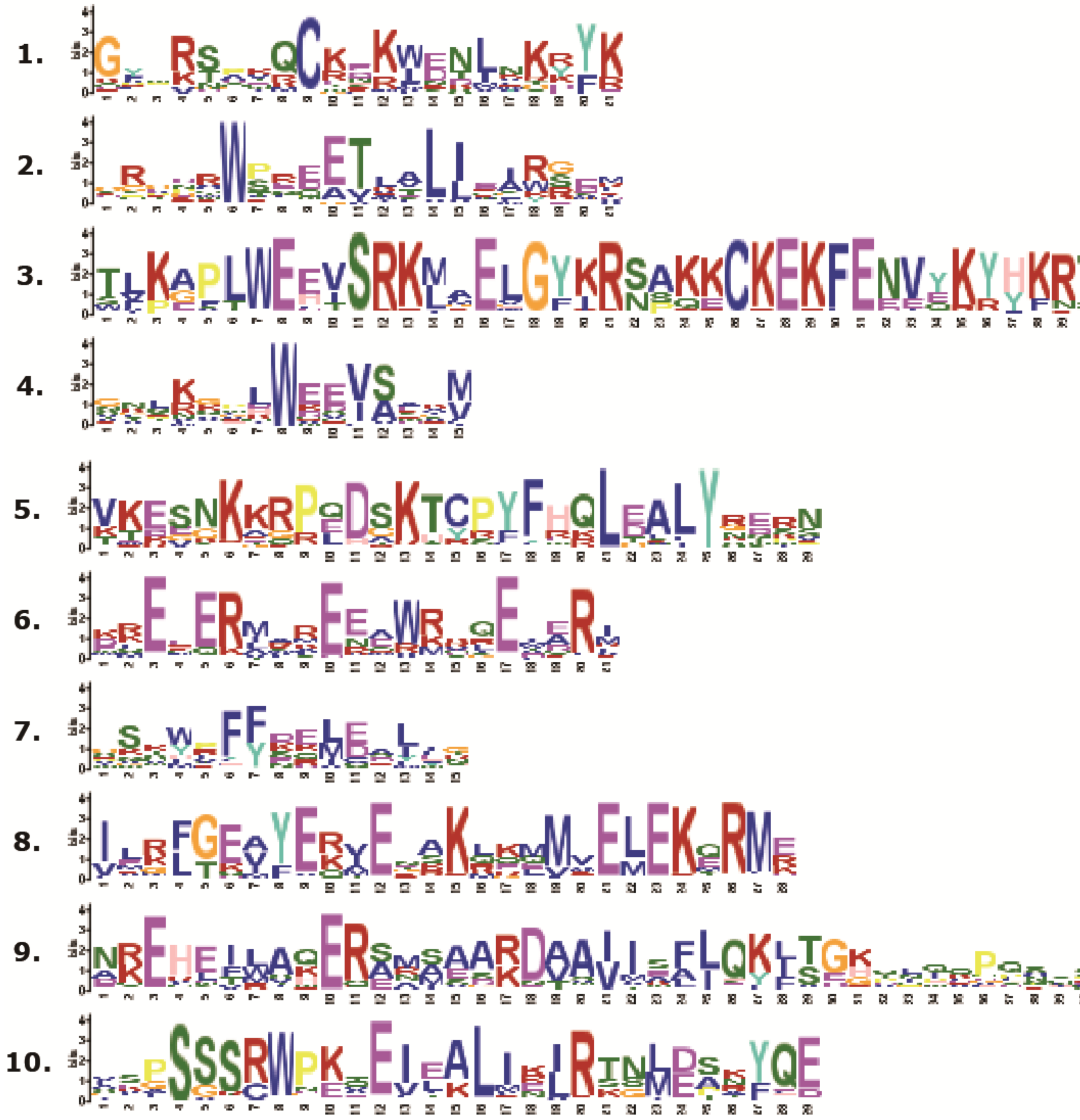

Supplementary Fig. 2 Multilevel Consensus Sequence and their logo of trihelix proteins as predicted by MEME program.

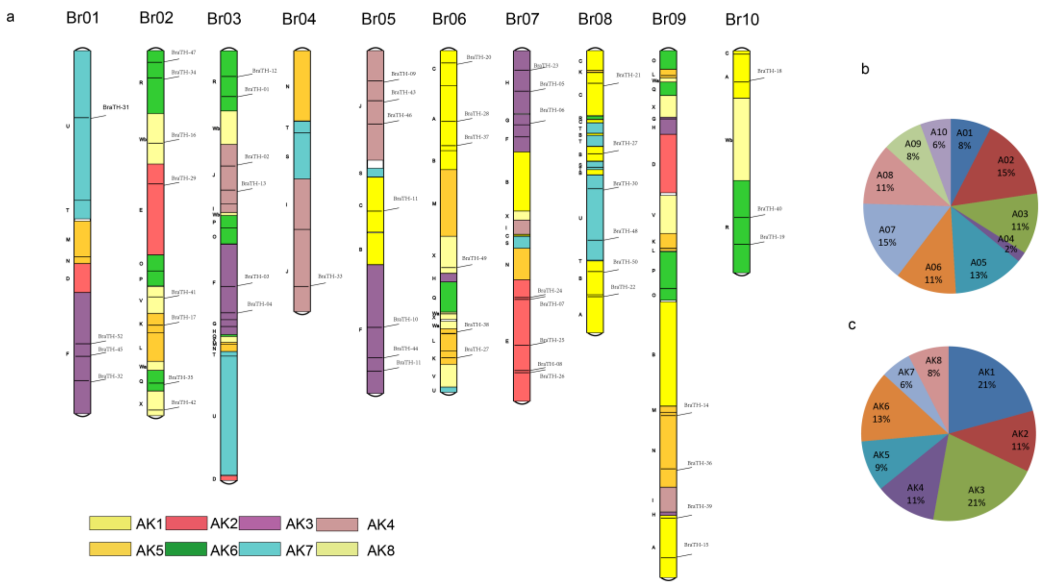

Supplementary Fig. 3 a Distribution of trihelix genes on 10 chromosomes based on the report of Cheng et al. (2013). **b** The percentages of trihelix genes on each chromosome are demonstrated by the *pie*. **c** The percentages of trihelix genes on each ancestral blocks are demonstrated by the *pie*.

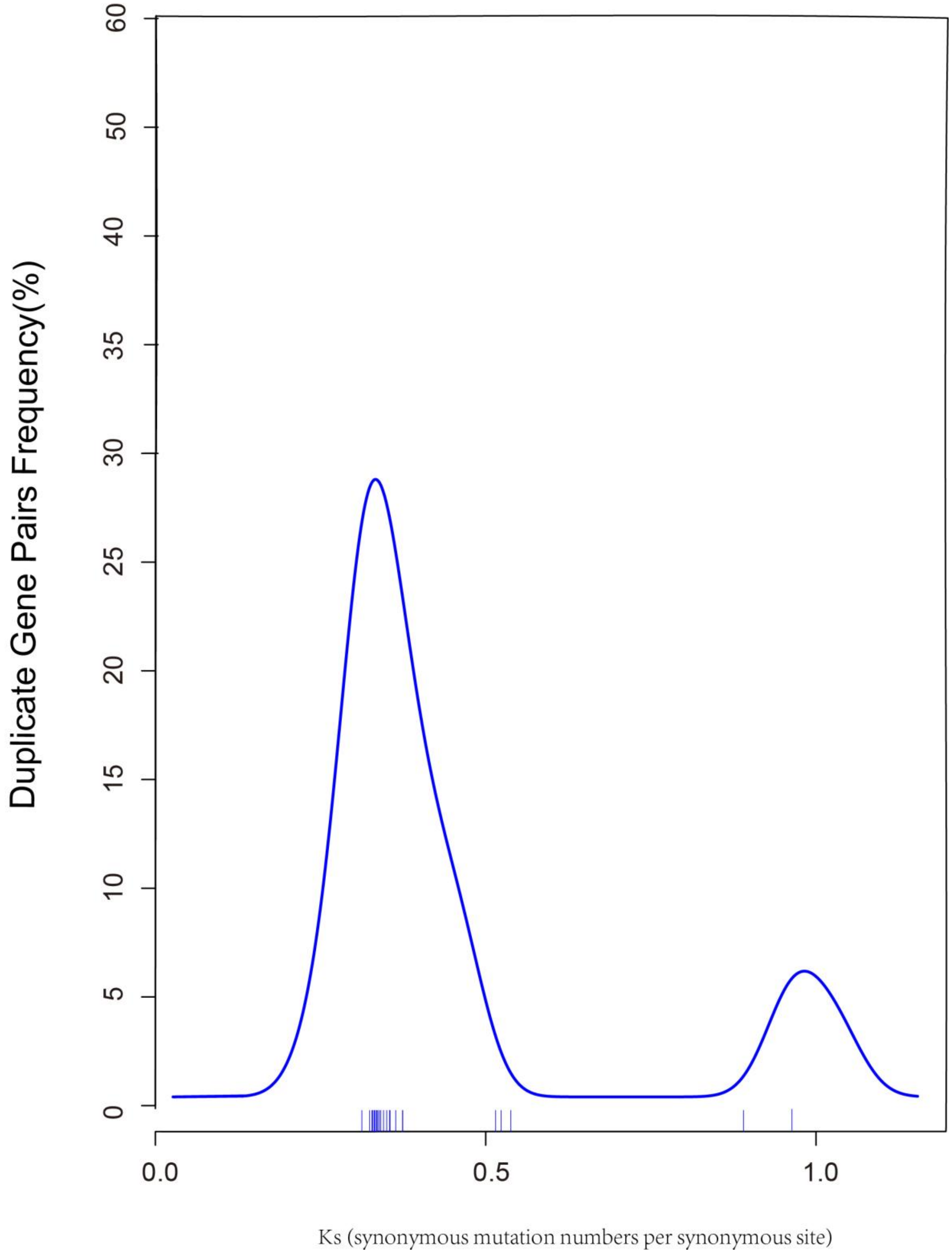

Supplementary Fig. 4 The distribution of the Ks values for *BraTH* duplicated genes.

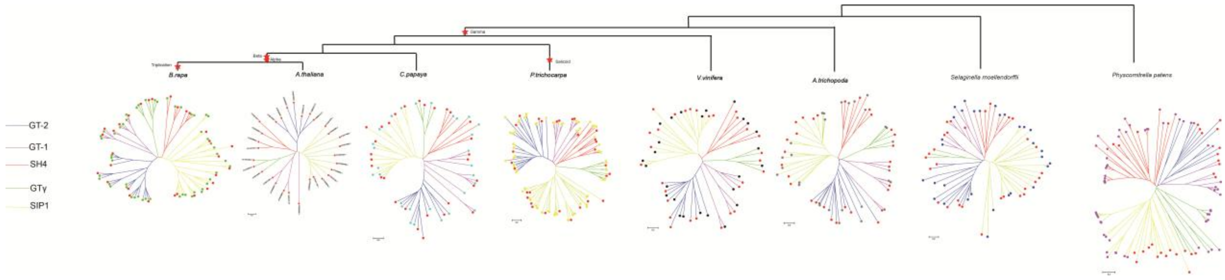

Supplementary Fig. 5 Information of trihelix gene family clades in *Brassica rapa*, *Arabidopsis thaliana*, *Carica papaya*, *Populus trichocarpa*, *Vitis vinifera*, *Amborella trichopoda*, *Phscomitrella patens* and *Selaginella moellendorffii*. The α, β, γ, and salicoid duplications and the *Brassica*-specific triplication are indicated on the branches of the trees according to the Plant Genome Duplication Database. To classify these trihelix genes, phylogenetic trees with trihelix genes for each of the 8 species by maximum likelihood using MEGA5, respectively.

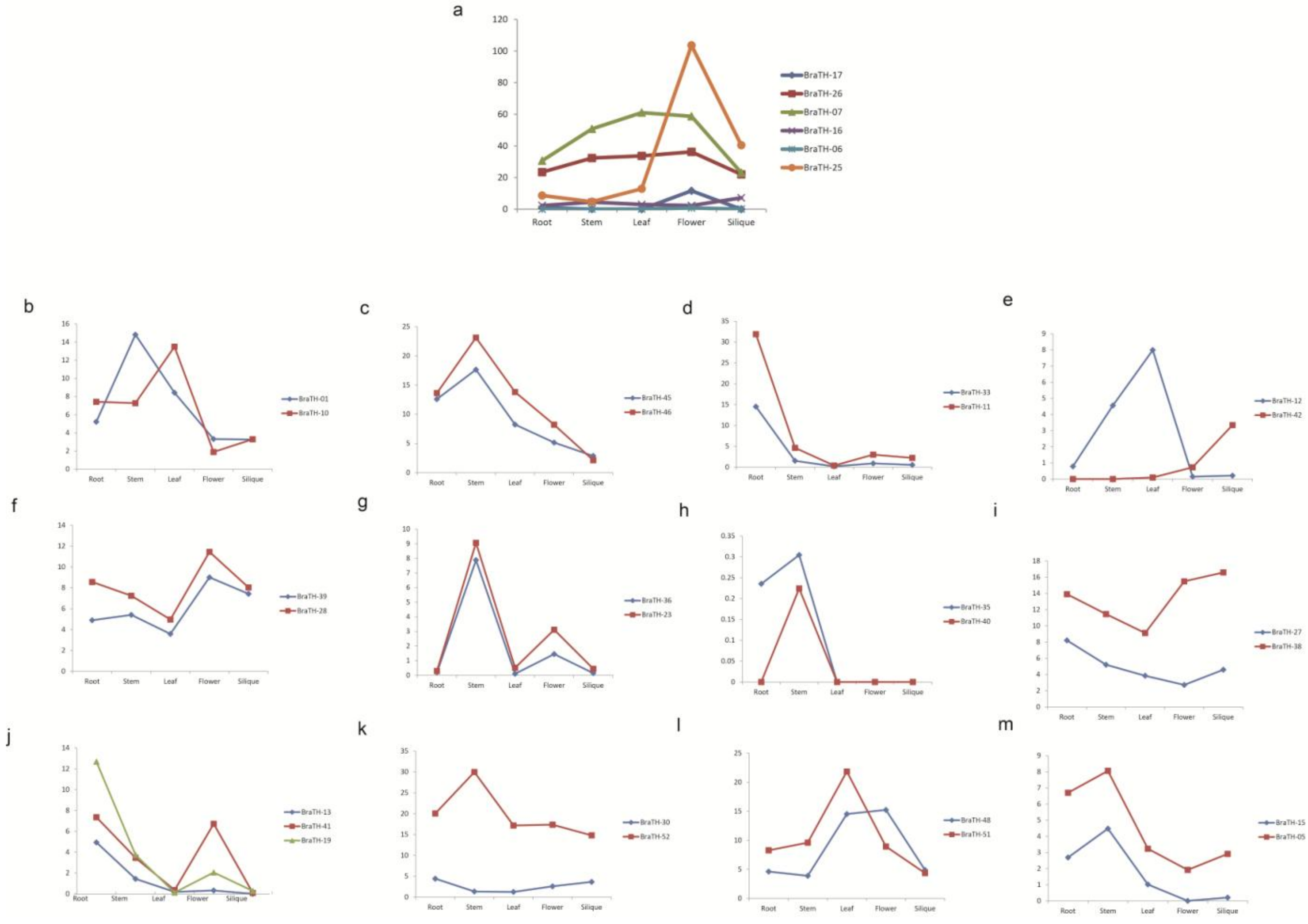

Supplementary Fig. 6 Comparison of the expression level in six tissues for duplication of trihelix genes.

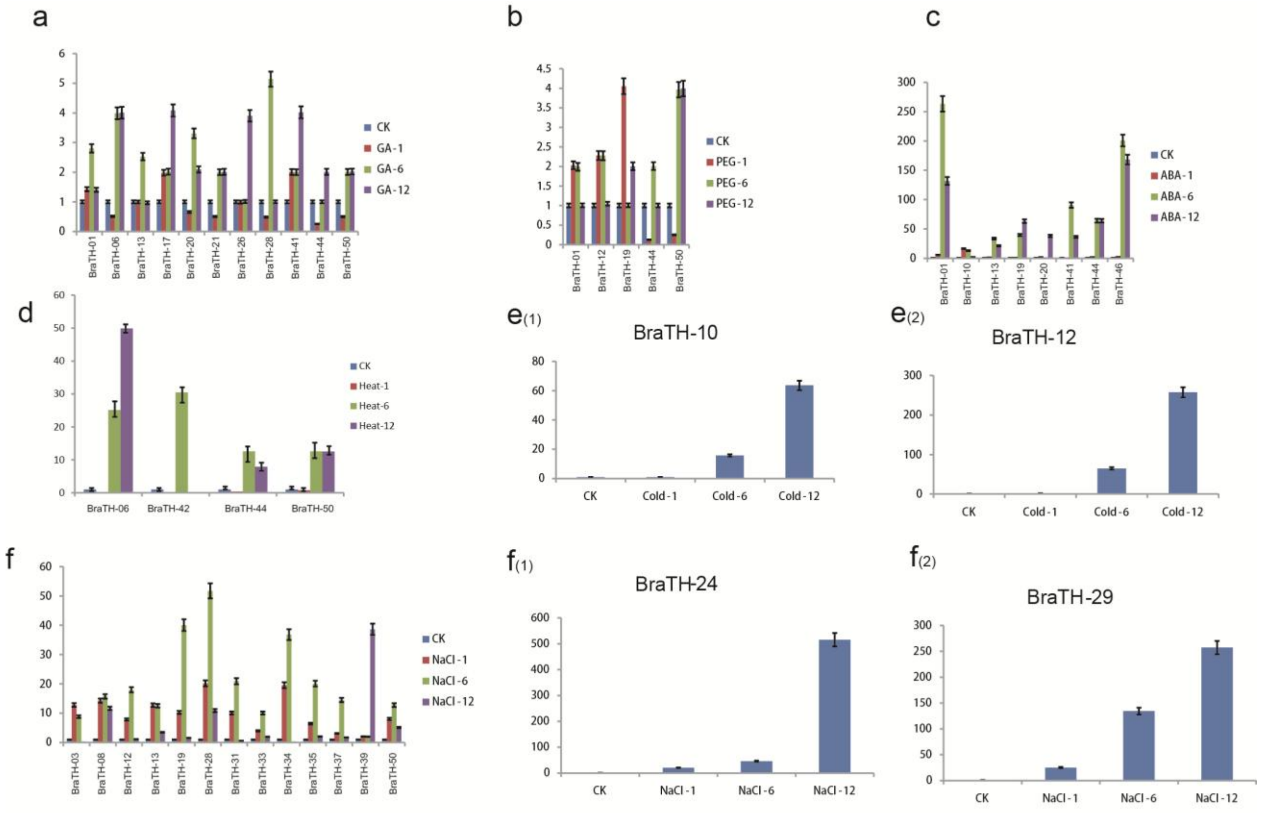

Supplementary Fig. 7 The relative expression ratio of trihelix genes under abiotic stresses. a GA; b PEG ; c ABA; d heat; e Cold; f Nacl; *Error bars* represent standard errors from three independent replicates.

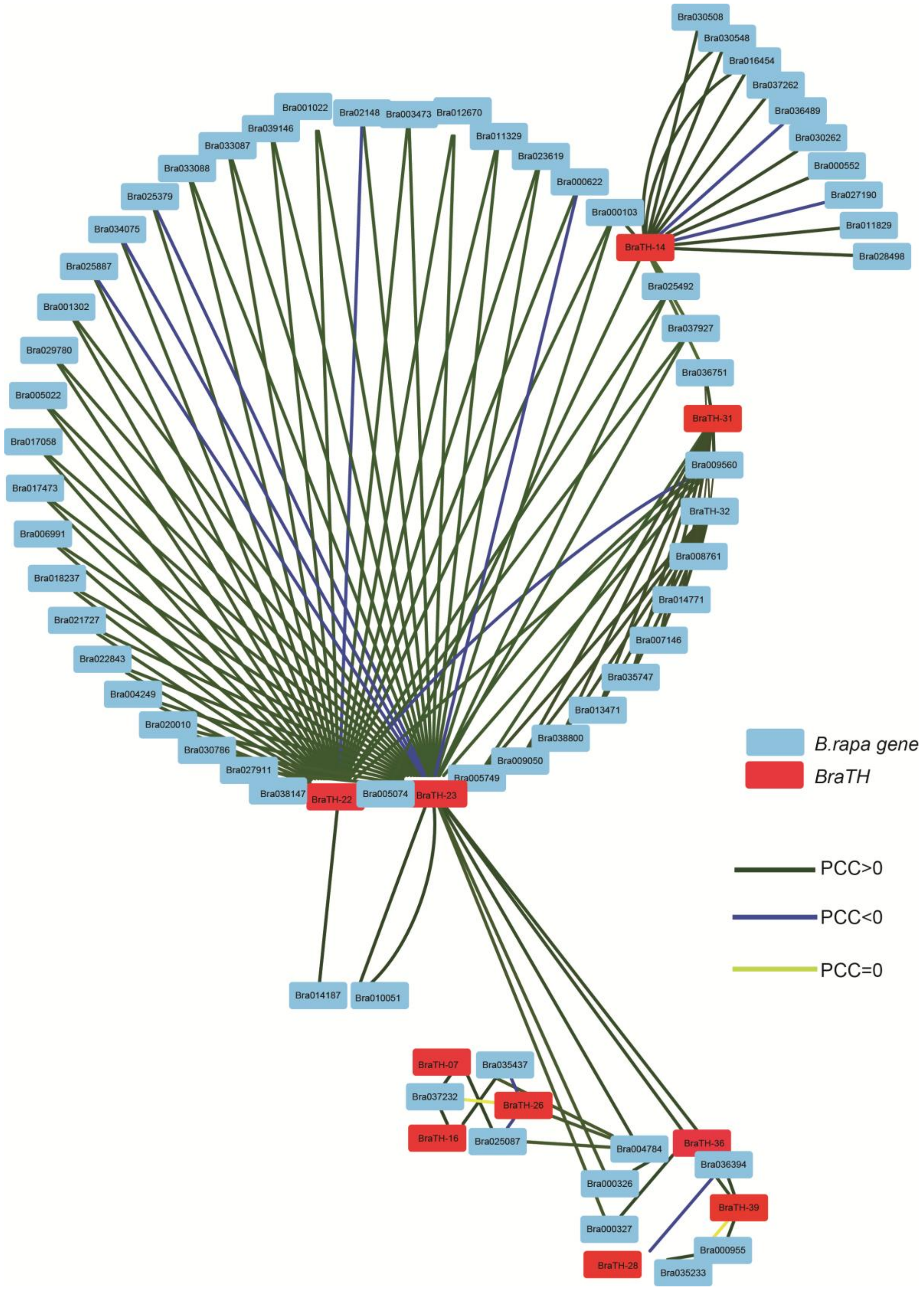

Supplementary Fig. 8 The interaction network of trihelix genes in *B.rapa* according to the orthologs in *Arabidopsis*. PCC Pearson correlation coefficient. The bule represent the Chinese cabbage genes, and the red represent the trihelix genes of Chinese cabbage
